# Supplementary material for: Genome-Wide Small RNA Sequencing Identifies MicroRNAs Deregulated in Non-Small Cell Lung Carcinoma Harboring Gain-of-Function Mutant p53
Source: Genes (Basel). 2019 Oct 28;10(11):852. doi: 10.3390/genes10110852 (PMC6895929; doi:10.3390/genes10110852)
Supplement: Supplementary file 1 [file genes-10-00852-s001.zip › genes-591077-SI/genes-591077-supplementary (1)/Supplementary information/Supplementary information.doc]

**Supplementary information**

**Genome-wide Small RNA Sequencing Identifies microRNAs Deregulated in Non-Small Cell Lung Carcinoma Harboring *Gain-of-Function* Mutant p53**

**Arindam Datta^1,^** ^±^**, Pijush Das^1,^** ^±^**, Sanjib Dey^1^, Sangeeta Ghuwalewala^1^, Dishari Ghatak^1^, Sk. Kayum Alam^1^, Raghunath Chatterjee^2,^ * and Susanta Roychoudhury^3,^ ***

**^1^**Cancer Biology and Inflammatory Disorder Division, CSIR-Indian Institute of Chemical Biology, 4, Raja S.C. Mullick Road, Jadavpur, Kolkata-700032, India. **^2^**Human Genetics Unit, Indian Statistical Institute, 203 B. T. Road, Kolkata 700108, India. ^3^Saroj Gupta Cancer Centre and Research Institute, Mahatma Gandhi Road, Thakurpukur, Kolkata 700063, India.

**Corresponding Author’s Name and Mailing Address**:

^*^(1) Susanta Roychoudhury, PhD, Saroj Gupta Cancer Centre and Research Institute, Mahatma Gandhi Road, Thakurpukur, Kolkata 700063, India. Phone: +91 033 2453 2781/82/83 (Ext 2118/2115), Fax: +91 033 2467 8002, E-mail: [susantarc@gmail.com](mailto:susantarc@gmail.com), [research@sgccri.org](mailto:research@sgccri.org).

^*^(2) Raghunath Chatterjee, PhD, Human Genetics Unit, Indian Statistical Institute, 203 B. T. Road, Kolkata 700108, India. Phone: 91-33-25753243, Fax: 91-33-24735197, E-mail: rchatterjee@isical.ac.in, [raghuchatterjee@gmail.com](mailto:raghuchatterjee@gmail.com).

± These authors contributed equally to this work.

**Supplemental Figure S1**

**Supplemental Figure S1. Quality control of small RNA sequencing data (A)** Agilent 2100 Bioanalyzer generated chromatograms of small RNAs isolated from H1299/EV and H1299/ p53^R273H^ cells. Percentage of enriched miRNA and miRNA/small RNA ratio are indicated**. (B)** Histograms showing length distribution of raw sequencing reads in individual samples.

**Supplemental Figure S2**

**Supplemental Figure S2. Regulatory networks of mutant p53^R273H^-regulated miRNAs.** Top three significant miRNA-mRNA networks and their associated functions are shown. Molecules shown in bold are present in our dataset. Red arrow indicates up-regulated and Green arrow indicates down-regulated miRNAs in presence of mutant p53^R273H^.

**Supplemental Figure S3**

**Supplemental Figure S3. Small RNA seq mapped reads alignment around miR-X region in Integrative Genomics Viewer (IGV).** Snapshot showing the alignment of the small RNA sequencing reads obtained from lung cancer dataset (GSE33858) to the genomic region (chr1:168,239,797-168,239,868; hg19) of miR-X.
